# Supplementary material for: Compassion as a guiding framework for the implementation of digital mental health interventions: An interview study with clients and professionals
Source: PLoS One. 2025 Oct 23;20(10):e0320710. doi: 10.1371/journal.pone.0320710 (PMC12548926; doi:10.1371/journal.pone.0320710)
Supplement: S1 File — (DOCX) [file pone.0320710.s002.docx]

**Interview guide for semi-structured interviews with (former) clients in mental healthcare**

Intro:

Today I am curious about your experiences with your treatment and what you find important in it. Your experiences as a client are important input for the research I am doing. That research is about what the treatment process in mental health care looks like, and what the needs are for therapists and clients. This way, the treatment could better match these needs in the future. At the end of the interview I can also tell you more about this.

In this interview we could of course go into depth about the complaints for which you were treated, but I am now particularly interested in the process of the treatment and what you thought of it, and different roles in the treatment of the client and therapist.

1. Would you first like to tell me briefly about what and where you were treated?
2. What did the treatment look like? What did it consist of?
   1. How was the contact with the therapist? How and how often was there contact?
   2. Were digital tools used, such as online modules with information and exercises, chatting, virtual reality, apps, in the treatment?
      1. If yes: How was this implemented/how was it introduced and then (not) used?
      2. What did you think of that approach?
   3. Have you used any other digital tools yourself to deal with your complaints?
3. Think back to when you first came in. Was it immediately clear what the treatment would look like? Or did that come a bit later? How was that determined?
   1. Did you discuss this with the therapist? Did the therapist make a proposal? Someone else? Or was it 'just' like that?
   2. What did you think of that approach?
4. How did you experience the treatment?
   1. What were the pleasant aspects of the treatment?
   2. What were the less pleasant aspects of the treatment?
5. What do you consider important in a treatment?
6. What do you need in a treatment?
7. I would now like to talk about the different people involved in the treatment and their roles. This mainly concerns the therapist and you as a client.
   1. What role did the therapist play in the treatment, and how did you feel about it? [To help you get started: was the therapist in charge? Did the therapist explain things? Did the therapist support you in the process?]
   2. What role did you play in the treatment yourself, and how did you feel about it? [Were you very involved in the treatment yourself, were you in charge, did you figure out how things worked, did you mainly follow the therapist]
   3. [If the client also mentions forms of eMental health in question 2] What role did [mentioned eMental health] play in the treatment? How did you feel about it? [Small or larger role, supportive, or really part of the treatment]
   4. What role did the environment play in your treatment? (Give examples: municipality, in the neighborhood, immediate circle of acquaintances/family/friends). How did you feel about that?
8. Are there any other things that you think could improve the treatment?
9. [If eMental health was not or only partially mentioned in question 2] > There are also other ways to complete or supplement a treatment. For example, by working with online exercises as a client, viewing and evaluating your stress levels or sleep quality with a ring or bracelet, using virtual reality to practice difficult situations, and so on. Would you see added value in these kinds of things where you use technology such as an app, VR, or smartwatch? Why (not)? And if so, in what way?
10. I have a few additional questions about how you experienced your treatment. Did you feel heard in the treatment? How did you experience that?
11. Did you feel during the treatment that it was okay that you had certain complaints or problems? How did you experience that?
12. Did you experience empathy during the treatment? How did you find that?
13. Did you feel during the treatment that the therapist could handle it when you were having a hard time? How did you experience that?
14. Did you feel during the treatment that the therapist wanted to help you with your complaints or problems? How did you experience that?
15. Finally, I have a special interest in compassion from my project. Compassion is about the awareness that someone is suffering, not judging someone else for it, empathizing, staying calm yourself, and wanting to alleviate this suffering. For example, a therapist can first ask questions and listen to get an idea of ​​your situation, empathize with you and then offer tools to alleviate the situation somewhat. What do you think of 'compassion', is this important to you in treatment? In what way?
16. In this research we look at the role that technology can play in supporting compassion in treatment. Is there anything else you would like to share?

Thank you for your participation!
