# Supplementary material for: Compassion as a guiding framework for the implementation of digital mental health interventions: An interview study with clients and professionals
Source: PLoS One. 2025 Oct 23;20(10):e0320710. doi: 10.1371/journal.pone.0320710 (PMC12548926; doi:10.1371/journal.pone.0320710)
Supplement: S2 File — (DOCX) [file pone.0320710.s003.docx]

**Interview guide for semi-structured interviews with professionals in mental healthcare**

First of all, I am curious about your job and what you find important in it.

1. Could you briefly tell me something about your work? What does a working day look like?
2. What kind of clients do you treat?
3. Why did you choose this job/field of work?
4. What motivates you to (continue to) do this work?
5. What are the difficult aspects of your job? (what costs energy)
6. Values ​​are things that you find important to strive for. What are important values ​​for you within your job in mental health care? [Write this down]

Now I would like to talk about the treatment.

1. How do you determine what a treatment will look like? (first open question, then further probes: e.g. frequency, duration, intensity, f2f or (also) at a distance)
2. What do you take into account? How do you do that, what considerations do you make?
3. Do you also use technology in your treatment, and if so, which one(s)? For example, email, chat, online modules, video calling, VR, wearable technology, etc. (I mean use for therapeutic purposes, so not email contact with colleagues)?
4. What do you take into account when using [previously mentioned technology]? How do you do that, what considerations do you make?
5. Are there any issues you encounter?
6. In what way is technology (eMental Health) introduced within your organization (or does this not happen)?
   1. How is it offered, with what reasons / motivation / thoughts of the organization?
   2. Do you also really notice that on the work floor?
7. What do you think of that approach of eMental Health? (first open questions, then ask if things are missing)
8. What do you think of that approach, when you look at the values ​​that you find important in your job [repeat values from question 6 to participant]?

Finally, in my project I have a special interest in the value of compassion. Compassion is about the awareness that someone is suffering, sympathy, and wanting to alleviate this suffering.

1. What do you think of this value, is it applicable within mental health care? Why (not)?

Those were my questions. Do you have any questions, or things that you would like to share for this project?

Thank you for your participation!
